# New Approach for the Construction and Calibration of Gas-Tight Setups for Biohydrogen Production at the Small Laboratory Scale

**DOI:** 10.3390/metabo11100667

**Published:** 2021-09-29

**Authors:** Caroline Autenrieth, Shreya Shaw, Robin Ghosh

**Affiliations:** 1 Institute of Biomaterials and Biomolecular Systems, Department of Bioenergetics, University of Stuttgart, Pfaffenwaldring 57, D-70569 Stuttgart, Germany; Shreya.Arun.Shaw@asu.edu (S.S.); robin.ghosh@bio.uni-stuttgart.de (R.G.); 2 School of Molecular Sciences, Tempe Campus, Mailcode 1604, Arizona State University, Tempe, AZ 85281, USA

**Keywords:** biohydrogen, purple bacteria, *Rhodospirillum rubrum*, dark photosynthesis, gas chromatography, calibration, H_2_-technology

## Abstract

Biohydrogen production in small laboratory scale culture vessels is often difficult to perform and quantitate. One problem is that commonly used silicon tubing and improvised plastic connections used for constructing apparatus are cheap and easy to connect but are generally not robust for gases such as hydrogen. In addition, this type of apparatus presents significant safety concerns. Here, we demonstrate the construction of hydrogen-tight apparatus using a commercially available modular system, where plastic tubing and connections are made of explosion-proof dissipative plastic material. Using this system, we introduce a gas chromatograph calibration procedure, which can be easily performed without necessarily resorting to expensive commercial gas standards for the calibration of hydrogen gas concentrations. In this procedure, the amount of hydrogen produced by the reaction of sodium borohydride with water in a closed air-filled bottle is deduced from the observed decrease of the oxygen partial pressure, using the ideal gas law. Finally, the determined calibration coefficients and the gas-tight apparatus are used for the analysis of simultaneous oxygen consumption and hydrogen production of the purple photosynthetic bacterium, *Rhodospirillum rubrum*, during semi-aerobic growth in the dark.

## 1. Introduction

Many cellular metabolic processes are intrinsically associated with gas production or consumption. The construction of a gas-tight apparatus for hydrogen production and measurement is not a challenge in an engineering environment. However, in the area of biohydrogen (bio-H_2_) production by algae, cyanobacteria and purple bacteria (for relevant reviews see [1,2,3,4,5]), the initial process development strategy is usually performed at the small laboratory scale [6,7,8,9,10,11,12], and many of the current developments are being undertaken by biologists, who often have little or no formal training in engineering, and are often stymied by the technical requirements for gas production and collection. Typically, training in the construction of tubed apparatus in a biochemical laboratory usually involves improvised connections between silicon tubing (which is prone to sparking due to static charging) and PTFE or silicon plugs, or to glass tubing. This type of apparatus (such as that described in a recent protocol [13]) is cheap, delivers results, but is not really hydrogen gas-tight, and presents significant safety concerns. In particular, the so-called EX-protection (i.e., the apparative measures necessary for *preventing* explosions and for *protecting* experimentalists against explosions) can only be achieved when the experimental setup is placed into an explosion proof hood. The technical alternative to silicone tubing, tailor-made all-metal connections are not only expensive, but are also difficult to construct without training in engineering or access to the appropriate tools.

One aspect of the present work is to demonstrate that a recently introduced modular EX-protected (the plastic material has been rendered highly conductive (1000 kOhm^−1^.m^−1^) by mixing with conductive graphite) construction system, originally conceived for chemical engineering applications, which is also extremely gas-tight to H_2_, can be easily modified for biological applications where the measurement of metabolic gases is an issue. The gas tight-assembly setups built with this material can be used outside a protective hood, which facilitates the use with larger volumes, commonly employed in growth experiments, and provides a straightforward path to up-scaling in an engineering environment.

For the accurate measurement of bio-H_2_, gas chromatography (GC) is the method of choice (see [14] for an extensive comparison of different methods for measurements of bio-H_2_ production. We note that the methods we present below are complimentary to those described in [14]). However, a significant problem for biologists in the bio-H_2_ area is the calibration of the GC for the measurement of gas production in closed vessels, such as bottles or shake flasks, commonly used for hydrogen production at the small laboratory scale. The problem here is that bio-H_2_ production from a microorganism in a closed bottle, almost inevitably leads to an increase in total pressure within the headspace to be sampled, which, without appropriate correction, can lead to significant errors when the gas composition is monitored by GC. In the method we present here, however, our closed, gas-tight, EX-protected setup uses a large gas reservoir to keep the total pressure nearly constant. Gas samples are measured with a GC, which has been calibrated beforehand using a simple calibration method (using only GC raw data and a knowledge of the gas laws), which is also described in the first part of our study. This method is now suitable for measurements of semi-aerobic growth regimes, where the consumption of O_2_ and the production of CO_2_ and H_2_ occur simultaneously, which is not measurable using the water displacement method [13], since an observed pressure change is not linear with the level of H_2_ production. Our method can easily be extended to samples of non-atmospheric pressure provided that gas dilution prior to measurement is performed (see below).

Finally, we present an application of our protocol, by analysing the gas (O_2_, H_2_ and CO_2_) uptake and production of the purple non-sulphur bacterium *Rhodospirillum rubrum*, grown using a high cell density medium that also induces the photosynthetic genes (including some responsible for H_2_ production) maximally under the dark conditions [15].

## 2. Results

### 2.1. Calculation of the Conversion Coefficients for the Determination of the Concentrations of O_2_, H_2_ and CO_2_ Directly from the GC Peak Area

**Figure 1:**
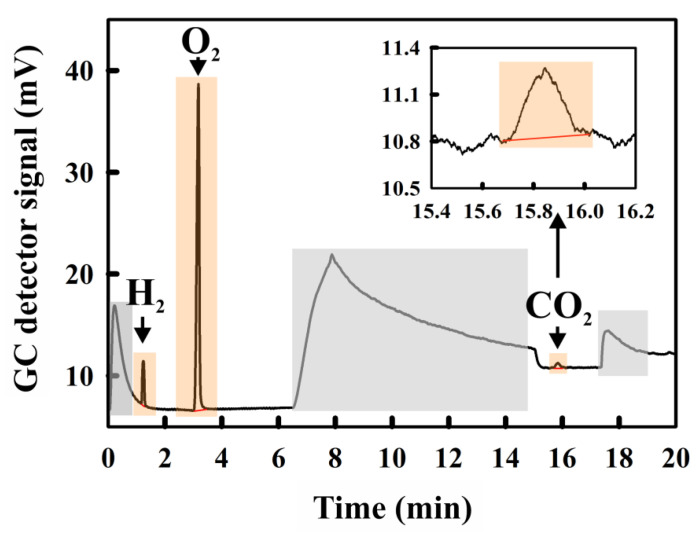
Gas chromatography chromatogram for a gas sample containing H_2_, O_2_, and CO_2_ (orange shaded regions), respectively. Inset: expanded view of the CO_2_ peak; grey shaded regions: the background output due to the injection artefact (0–1 min) due to transient mixing of the N_2_ carrier gas with the sample, the temperature ramp (start at 6.5 min), and residual dirt in the column (17 min).

A typical GC-profile of a H_2_-containing gas sample is shown below (Figure 1). The assignment of peaks was performed as described in Section 4.

The conversion of the peak signal area (in mV·min) into an absolute gas concentration can be demanding. One possibility is to employ a reference sample with a defined gas mixture of known concentrations, usually obtained from a gas cylinder. However, in this case, the experimental difficulty is to correct for the higher pressure in the gas cylinder, which is released in the sample measuring loop. In addition, this method generally requires the availability of commercial gas mixtures, which can be costly to maintain. In this work, we demonstrate an alternative method, whereby, using only ambient air and a simple H_2_-generating system, the absolute concentrations of O_2_, H_2_, and CO_2_ can be determined directly from the GC profile by a straightforward application of the ideal gas equation.

#### 2.1.1. The Reference Value: The GC-Conversion Coefficient for O_2_

For determination of the O_2_ conversion coefficient, ambient air samples were used. Atmospheric air has a near-constant gas composition and contains 78.10 mol % N_2_, 20.95 mol % O_2_, small amounts of argon (approx. 0.9 mol %) and other (noble) gases, and 0.03–0.05 mol % CO_2_. Thus, the GC-peak area obtained for O_2_ can be converted directly into the molar O_2_ concentration by applying the ideal gas equation:

(1) $$P_{O_{2}}V_{L}=n_{O_{2}}R\;T$$

where P_O2_ is the partial pressure of O_2_, V_L_ is the volume of the sample loop (1 mL), n_O2_ is the molar number of O_2_ in the sample, R is the gas constant (8.314 J·mol^−1^·K^−1^) and T is the absolute temperature of the sample.

To obtain a reliable value for this coefficient, a total of 22 O_2_ peak area (PA) measurements were performed over a period of 4 days. For each measurement, the ambient temperature (20.0–21.3 °C) was measured with a digital thermocouple and the atmospheric pressure (0.0993–0.1037 MPa) was obtained from the online campus weather station.

From these measurements we calculated the O_2_ conversion coefficient:

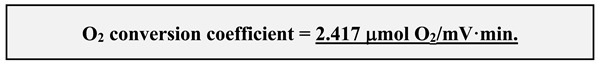



The standard deviation was 0.054 µmol/mV·min, i.e., a 2.2% error.

#### 2.1.2. Determination of the GC-Conversion Coefficient for H_2_

We have employed the well-known hydrolysis of sodium borohydride (NaBH_4_) to generate defined amounts of H_2_ in a closed system. The stoichiometry of the reaction (in the presence of excess H_2_O) is given by Equation (2):

NaBH_4_ + 2 H_2_O ⇒ NaBO_2_ + 4 H_2_ (2)

Note that, for clarity, we only show the major reaction of NaBH_4_ with excess H_2_O here. The hydrolysis of NaBH_4_ has been considered in detail elsewhere [16,17].

**Figure 2:**
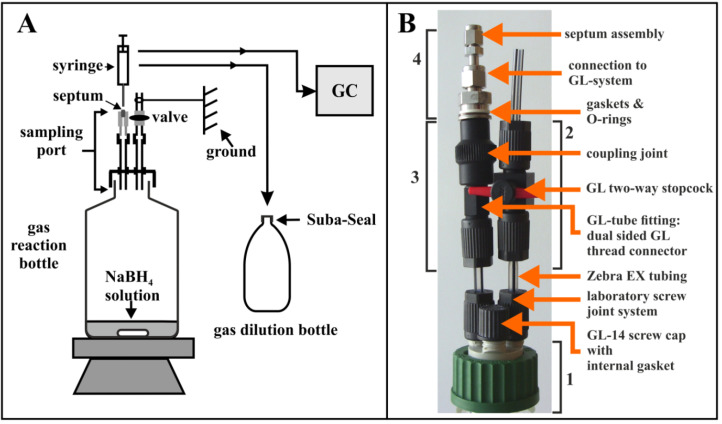
(**A**) Schematic diagram of the gas-tight bottle assembly used for the production of H_2_ from defined amounts of NaBH_4_. Gas sampling was performed by using a home-made sampling port assembly containing a gas-tight septum. In this Figure, a “gas dilution bottle” (sealed with a Suba-Seal^®^ cap) is also shown, which can be used to dilute pressurized gas samples to atmospheric pressure (see Materials and Methods). (**B**) Details of the gas-tight sampling apparatus: (1) GL-45 cap with 3 GL-14 threaded distributors on its head. The unused port is sealed with a GL-14 screw-cap; (2) an assembly with a connection to an external tubing, which can be used for evacuating the bottle and for flushing the bottle with N_2_ or other (standard) gas mixtures; (3) the connecting parts between the bottle and (4) the septum assembly containing the gas-tight septum.

The H_2_-producing reaction was performed in a 1 litre bottle, which was closed with a home-made gas-tight cap assembly incorporating a septum for gas sampling. We used connections and tubings made of a special conductive plastic material. This material is particularly advantageous for small-scale laboratory setups in that the parts made from it are not only designed to be H_2_-tight but also incorporate chemically inert but conductive material which dissipates static charge, thereby mitigating the danger of explosion. Figure 2 shows our experimental gas-tight setup for H_2_ production in a closed bottle. More details can be found in Section 4 and in Supplementary Figure S1 and Supplementary Table S1.

For the initiation of the H_2_-producing reaction, a NaBH_4_ pellet (0.23 g NaBH_4_ pellet (doped with 10% (*w*/*w*) Co^2+^ catalyst)) was placed in 40 mL H_2_O in the gas-tight bottle assembly, and the cap closed immediately. According to equation (2), the hydrolysis of 0.23 g NaBH_4_ dissolved in excess H_2_O should yield 21.89 mmol H_2_. This amount of H_2_ will generate an excess pressure of 0.47 atm (at 20 °C, in the total gas volume of 1.11 L), assuming that the reaction goes to completion. The total pressure in the closed bottle is described by Dalton’s law:

(3) $$\mathrm{Air}\;\mathrm{in}\;\mathrm{bottle}\;(\mathrm{before}\;H_{2}\;\mathrm{generation}):\;P_{T}=P_{O_{2}}+P_{CO_{2}}+P_{N_{2}}+\sum_{i}P_{i}$$

(4) $$\mathrm{Air}\;\mathrm{in}\;\mathrm{bottle}\;(H_{2}-\mathrm{generation}\;\mathrm{initiated}):\;P_{T}^{H}=P_{O_{2}}+P_{CO_{2}}+P_{N_{2}}+\sum_{i}P_{i}+P_{H_{2}}$$

where *P_T_* and $P_{T}^{H}$ are the total pressures in the bottle before and after H_2_ generation, respectively, and P_O2_, P_CO2_, P_N2_, P_H2_ refer to the partial pressures of the individual gases. $\sum_{i}P_{i}$ reflects the sum of the partial pressures of all other, minor components in air (e.g., argon and other noble gases).

**Figure 3:**
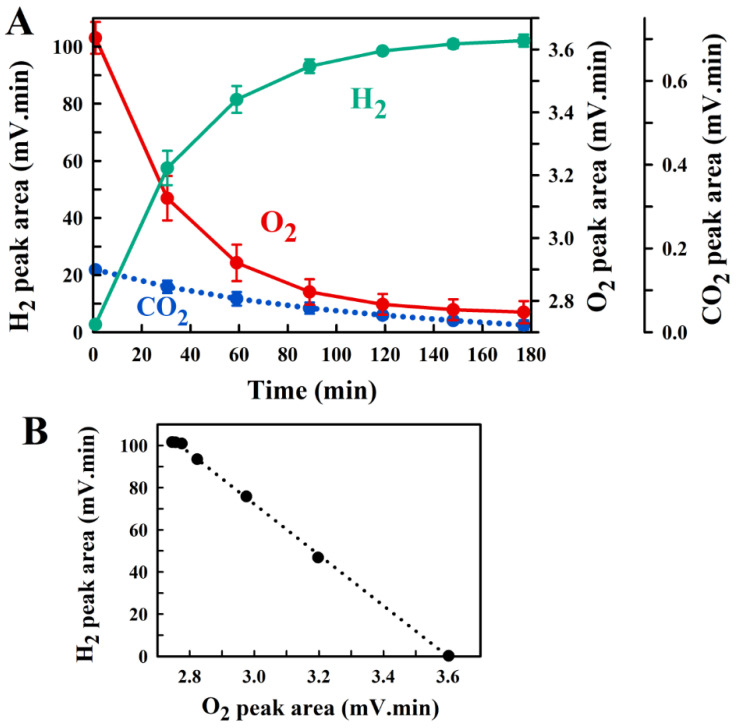
(**A**) The measured peak areas of the GC-chromatogram obtained in the NaBH_4_-H_2_ evolution experiment. Average values and the respective standard deviations (error bars) from four different experiments are shown. The zero time point refers to the time after addition of the NaBH_4_ pellet to water. The reaction is essentially complete after 150 min. The plateaus for H_2_ and O_2_ confirm the expectation that their reaction at room temperature is negligible for the time of the experiment. In (**B**) the linear correlation between increase of the H_2_ peak area and decrease of the O_2_ peak area is shown.

Now, when a 1.5 mL sample is extracted from the gas-tight bottle, the gas in the syringe is maintained at the sampling pressure. However, when this sample is injected into the sample loop, the total gas pressure ($P_{T}^{H}$ in the syringe) is instantly released to atmospheric pressure (*P_T_*), but now the relative partial pressures of the individual gases will be reduced compared to the initial values obtained prior to H_2_ generation, i.e.,:

(5) $$P_{T\;}\left(air+H_{2}\right)=P_{O_{2}}^{\prime }+P_{CO_{2}}^{\prime }+P_{N_{2}}^{\prime }+\sum_{i}P_{i}^{\prime }+P_{H_{2}}^{\prime }$$

where the P_i_′ values for O_2_, CO_2_, and N_2_ are less than their initial values measured prior to H_2_ generation. Thus, the gases measured at increasing time points after the initiation of H_2_ generation should show a linear negative correspondence between the PA(H_2_), due to H_2_ production, and the PA(O_2_), due to the suppression of the P_O2_ due to excess H_2_ partial pressure. A similar decrease of the CO_2_ PA should also be observed. Figure 3A shows the result of a typical NaBH_4_-H_2_ production experiment, and confirms the expectations (Figure 3B):

Using Dalton’s law we can show that:

(6) $$P_{T}^{H}=\gamma P_{T}$$

where:

(7) $$\gamma =\frac{PA\left(O_{2}\right)}{PA\left(H_{2}+O_{2}\right)}$$

where PA (O_2_) and PA (H_2_ + O_2_) are the peak areas of O_2_ measured before and after H_2_ generation, respectively.

Determination of the γ value allows the excess pressure (P_H2_) due to H_2_ in the closed bottle to be calculated:

(8) $$P_{T}^{H}-P_{T}=P_{H_{2}}$$

After sampling, the high pressure $P_{T}^{H}$ is maintained in the gas-tight syringe. Thus, the number of moles H_2_ per 1 mL in the syringe can be calculated by using the ideal gas equation:

(9) $$n_{H_{2}}=\left(P_{H_{2}}\cdot 1\;ml\right)\;/\left(RT\right)$$

After injection into the sample loop, the pressure is released to *P_T_* and consequently, the amount of moles H_2_ in the 1 mL sample loop is reduced to:

(10) $$n\prime_{H_{2}}=\gamma^{-1}\cdot n_{H_{2}}$$

The H_2_ conversion coefficient is then calculated by dividing n′_H2_ by the H_2_ peak area obtained in the GC run. (See Section 4 for the justification of Equations (6)–(10)).

Four different experiments for determination of the H_2_ conversion coefficient were performed. During those experiments, in total 17 GC-measurements were made and the peak areas of H_2_ and O_2_ obtained, from which the following average H_2_ conversion coefficient for the GC chromatogram H_2_ peak area was calculated:

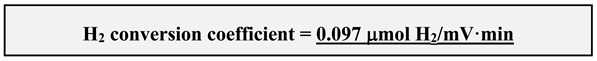



The standard deviation was 0.002 µmol/mV·min, i.e., a 1.8% error.

#### 2.1.3. Determination of the GC-Conversion Coefficient for CO_2_

Whereas measurements of air are of sufficient accuracy for the determination of the O_2_ conversion coefficient, the determination of a GC-conversion coefficient for CO_2_ is not possible with air measurements, since the CO_2_ peak is small and shows a very high variation from measurement to measurement. In 22 GC-measurements of 1.5 mL air, the CO_2_ peak varied between 0.0975 mV·min and 0.3661 mV·min (average: 0.2187 mV·min, standard deviation: 36.9%).

Therefore, a commercially supplied standard H_2_/CO_2_ gas mixture (2 vol. % CO_2_, 6 vol. % H_2_, and 92 vol. % N_2_) was used for CO_2_ calibration and to check the H_2_ conversion coefficient obtained by using the NaBH_4_-method (see Section 4 for sampling details).

In the event, we observed that the H_2_ conversion coefficient derived from the H_2_/CO_2_ standard (0.088 ± 2.8% μmol H_2_/mV·min) was consistently lower (about 9%) than the value calculated from ideal gas laws. However, we were able to show that of the 9% error, 3% is contributed by the syringe gas sampling error, which leaves only a 6% discrepancy between the calculated and measured values. We consider this error to be acceptable for most practical purposes.

The average CO_2_ conversion coefficient using the standard gas mixture was 0.240 ± 3.1% μmol CO_2_/mV·min.

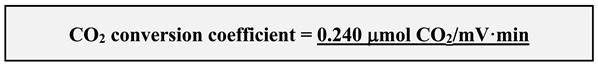



### 2.2. Experimental Setup for Semi-Aerobic Growth of a H_2_ Producing Photosynthetic Bacterium, R. Rubrum

In the following, we will describe an application example for the use of the gas-tight components for EX-protection and the determined GC-conversion coefficients described above. Usually, for H_2_-production with photosynthetic microorganisms including algae as well as purple bacteria, the cultures have to be shifted to anaerobic conditions before H_2_-production can occur [6,7,8,9,10,11]. In those setups, monitoring the H_2_-production phase is relatively easy, since H_2_ is the only variable gas component, and thus, a pressure increase can be correlated to the amount of H_2_ produced.

In bioreactor setups where organisms are grown in a mixed metabolic regime (usually some combination of anaerobic and aerobic metabolic modes [15,18], see [19] for more examples using different phototrophic bacteria) the profiles of gas evolution and consumption can more complicated.

Here, we demonstrate our method by analysing the gas utilisation and production of the purple photosynthetic bacterium, *R. rubrum*, under semi-aerobic (O_2_-limited growth, see below for a more detailed discussion of the relevant physiology), “dark photosynthetic” growth conditions. In *R. rubrum* (as for other photosynthetic organisms) H_2_ is released when “photosynthetic” anaerobic metabolism produces an excess amount of reducing equivalents, which is the case when fructose or pyruvate are one of the carbon substrates [18,20]. We have shown previously that under conditions of semi-aerobic growth (P_O2_ < 0.3% [18,21]) in a special culture medium, M2SF medium, that *R. rubrum* will maximally express photosynthetic genes that are normally produced under anaerobic photosynthetic conditions [15]. This growth regime we define as “dark photosynthetic”, since all photosynthetic gene regulatory mechanisms are in place, albeit in the absence of light. We note in passing that the semi-aerobic regime (which is determined by the number and amounts of cytochrome oxidases present) is different for other photosynthetic bacteria. Thus, for *Rhodobacter capsulatus*, the “semi-aerobic” growth regime causing photosynthetic membrane expression, is already observed at P_O2_ < 8% [22]. The transition from aerobic to “dark photosynthetic” semi-aerobic growth is causal to gene expression of specialized photosynthetic membranes, which are comprised of about 50% light-harvesting complexes. Since the absorbance at 882 nm is exclusively due to the light-harvesting complexes, the ratio A_882_/A_660_ is a diagnostic parameter for the level of photosynthetic membranes/cell. In a typical growth experiment, below cell densities corresponding to an A_660_ (4 mm path-length) of about 0.2, the genes required for photosynthesis (which include those for photosynthetic membrane production) are repressed by O_2_. At high A_660_ values, the O_2_ consumption by the cells is so high that the local (liquid phase) P_O2_ falls below 0.3%, and photosynthetic genes are expressed. If aerobic M2SF cultures are inoculated with cells from fully grown semi-aerobic cultures, where the A_882_/A_660_ ratio is high, photosynthetic membrane expression is initially arrested and the A_882_/A_660_ ratio decreases until the cell density reaches an A_660_ (4 mm) of 0.2, whereupon the A_882_/A_660_ ratio begins to rise. The result is a noticeable trough in the A_882_/A_660_ profile, which indicates the transition to semi-aerobic growth. Under normal M2SF growth conditions, the H_2_-producing nitrogenase is completely repressed by the high levels of ammonia in the medium [23,24]), so that H_2_ can only be produced by the action of the formate-hydrogen lyase [20,25,26], or the reversible hydrogenases [23,27], both of which are reversibly inhibited by O_2_.

**Figure 4:**
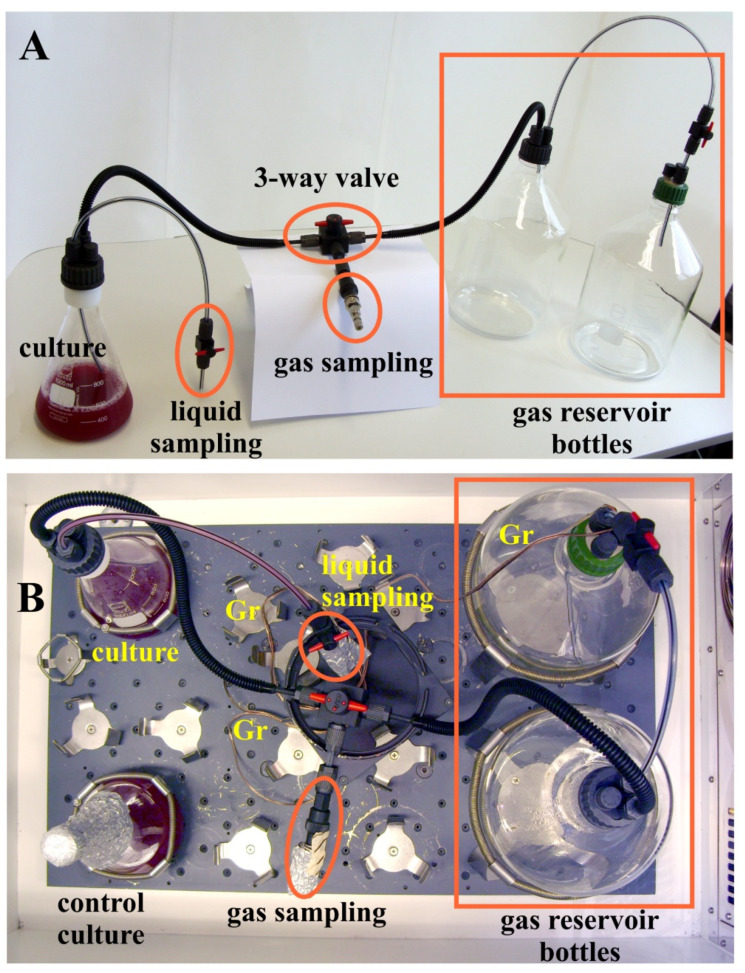
(**A**) The experimental setup for semi-aerobic incubation of *R. rubrum* using gas-tight and explosion-proof tubing and connections. (**B**) shows the positions of the bottles and flasks in the shaker during the growth experiment. The copper wire (Gr) used for grounding is also visible. The control culture (bottom left) is also shown. The growth experiment was performed in the dark (in a closed, light-tight shaker) at 30 °C.

At present, the dynamics of the reciprocal O_2_ utilisation and H_2_ production in semi-aerobic cultures of *R. rubrum* growing in M2SF medium have not been characterized. Here we use our method to conduct the first analysis of this effect. However, the difficulty in establishing an experimental design for this purpose is that, within a closed setup, the culture needs to access a large quantity of O_2_ for proper growth, and the H_2_ produced has to be collected for further analysis. Thus, in our initial setup (Figure 4), a one litre culture flask containing 524 mL of a *R. rubrum* culture was connected to two 5 litre bottles, which were used both as O_2_-reservoir and collection bottles for the H_2_-gas produced. The gas-tight setup incorporated switching valves for sampling of liquid and gas samples without modifying the gas composition as well as the sterility of the chamber during measurement.

**Figure 5:**
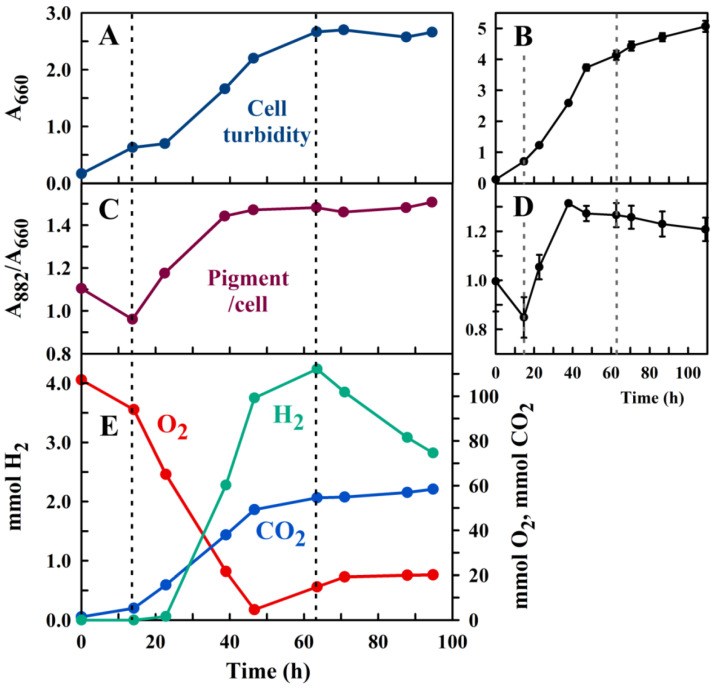
The *R. rubrum* growth experiment. (**A**,**B**) The absorption at 660 nm (A_660_, calculated for 1 cm path-length), reflecting cell growth of a single closed culture during the H_2_ production experiment (**A**) and the averaged data of three independent (open) control cultures (**B**). (**C**,**D**) The ratio A_882_/A_660,_ reflecting the expression of photosynthetic membranes of a single closed culture (**C**) and the averaged data of three control cultures (**D**). The (**B**,**D**) panels (which show also the standard deviations of the measurements) have been included to show the reproducibility of the growth experiment. (**E**) GC measurements of gases in the total 12.5 litre gas reservoir of the closed hydrogen producing culture (see Section 4). The values shown are calculated amounts per litre culture. The first dashed line (at 13.8 h) shows the commencement of semi-aerobic growth, the second dashed line (at 63.3 h) indicates the establishment of the stationary growth phase (see main text).

The results of a typical M2SF growth experiment in a 504 mL culture, using a 20 mL inoculum, are shown in Figure 5.

The time course of the experiment can be divided into 3 phases (separated by dashed lines in Figure 5), which reflect different physiological modes of the *R. rubrum* culture. At the start of the experiment, the culture medium is saturated with O_2_ (in air), and the gas reservoir contains O_2_ at the normal atmospheric concentration. During the first 14 h of cell growth, O_2_ in the medium is rapidly consumed, leading to the establishment of the semi-aerobic growth regime (at a P_O2_ < 0.3 [21]), which is indicated by the trough in the A_882_/A_660_. At this point, photosynthetic genes are induced (the “dark-photosynthetic” growth regime), reflected by the rise of the A_882_/A_660_ values. H_2_ accumulation starts approximately 9 h later (at a gas-phase P_O2_ ~ 13%) and reaches a peak at 63 h, after which the cells enter the stationary phase. In the stationary phase, all the fructose in the medium has been consumed, as indicated by measurements of fructose concentrations in the culture medium supernatant (data not shown). In this last phase, the depletion of cellular reducing equivalents can be partially compensated by the uptake of H_2_, mediated by the reversible H_2_ase activity. The apparent rise of O_2_ after the time point 47 h is an artefact due to the fact that in the late phase of growth, H_2_ and CO_2_ are consumed, which leads to a lower pressure than atmospheric, which is maintained in the gas-tight syringe after sampling. Thus, when the sampled gas is injected into the GC sample loop, air will enter the GC loop, thus (artefactually) raising the effective partial molar volume of the measured gases over their true values in the gas-tight syringe. We note that *R. rubrum* does not produce O_2_ under any growth conditions. In future works, an external digital manometer may be used to calculate the true gas concentrations for the late growth phase.

The final cell density of the H_2_-producing closed culture was only approximately half of that which was achieved by the control cultures (Figure 4B and Figure 5B) which had been closed with a standard cotton wool stopper, thereby allowing unhindered gas exchange with the atmosphere. However, the final pigment content (A_882_/A_660_) was 1.2-fold higher in the closed setup compared to the control cultures (compare panels in Figure 5C,D). Both the lower cell density and higher A_882_/A_660_ of the closed system indicated that this setup was O_2_-limited when compared with the control cultures. The possibility that the tubing connecting the sampling port to the reaction vessel is too thin to allow efficient gas exchange is unlikely, as separate measurements of gas exchange (using CO_2_ as a reference gas) showed that equilibration between culture and gas reservoirs occurred well within the time increment (about 12 h) of sampling (data not shown). However, since the solubility of O_2_ in water is relatively low (see [28] for a detailed discussion of O_2_ availability in microaerobic cultures), the local O_2_ concentration in the culture dropped below the limit that is needed for optimal growth.

The problem of O_2_ limitation might be circumvented by leaving the “closed” setup in an “open” state (i.e., freely accessible to air) for the first 24 h where no significant H_2_-production was observed, but where the A_660_ values of the control and closed culture already started to diverge. Additionally, the gas reservoirs could be exchanged during the experiment and a ventilation system installed inside the gas reservoir bottles, or, alternatively, a third 5 litre reservoir bottle could be connected to the setup. These optimisation possibilities will be studied in future work.

## 3. Discussion

In this work, we have established and calibrated a gas-tight and explosion proof setup for H_2_-production at the small biological laboratory scale, involving cultures of around 0.5–2 litres.

The use of H_2_ produced by the reaction of NaBH_4_ with H_2_O described in this study, together with the application of ideal gas laws, is an easy and cheap way to calibrate a GC without the use of commercial standard (often pressurized) gas mixtures, and can provide a starting point for more sophisticated experimental studies. In particular, the response of the GC detector from very low to high concentrations of H_2_ can be deduced in a single experiment. However, since the sampling septum geometry of the H_2_-generating system may be slightly biased by the accumulation of H_2_ at the top of the vessel over time, for very precise measurements it is advisable to employ a commercial H_2_-CO_2_ gas standard, to standardize the H_2_ detector response for a reference H_2_ concentration.

We are aware of course, that all the aforementioned gases are not strictly ideal in behaviour. However, at the low gas concentrations usually relevant for biological systems, the necessary corrections (employing the known virial coefficients) for real gas behaviour are negligible (see below). We also note that the quantitative effect of increased hydrogen partial pressures upon H_2_ production in closed vessels has also been considered previously for small (15 mL) closed anaerobic cultures of *Chlamydomonas reinhardtii* [29].

Throughout the work described here, a special emphasis was laid on the fact, that a self-built (non-technical) setup for biological H_2_ production still has to fulfill the demand for both gas-tightness (and therefore reliability of the results) and safety for the experimentalist, which is not possible, when silicon tubing is used. Here, we used special conductive plastic parts and metal parts for the assembly of gas-tight setups (see Section 4 and also the Supplementary Material for material sources). Our construction design could also be useful for adaptation to the small technical scale. The constructions shown here are both modular and robust and well-suited to biological work. Also the system does not utilize silicon tubing, which appears to be almost universally employed for small-scale bio-H_2_ production experiments. In fact, although silicon tubing is cheap, it is not really H_2_-tight and cannot be grounded, and is therefore highly inferior for bio-H_2_ experiments.

Finally, we have demonstrated the applicability of the method to a real bio-H_2_ production experiment, involving the purple photosynthetic bacterium, *R. rubrum*. We showed that the reciprocal dynamics of O_2_ consumption and H_2_ production as well as the net behaviour of CO_2_, under conditions of semi-aerobic growth, can be reliably followed, and are consistent with previous observations [18] and known biological mechanisms of H_2_ production in *R. rubrum*.

## 4. Materials and Methods

### 4.1. Chemicals and Materials

In general, chemicals were obtained from Sigma-Aldrich/Merck (Darmstadt, Germany), Roth (Karlsruhe, Germany), Häberle (Lonsee-Ettlenschieß, Germany), or VWR/Avantor (Darmstadt, Germany) and were of analytical (or higher) grade. Ultrapure water was taken from a Synergy^®^ UV water purification system (Millipore/Merck (Darmstadt, Germany)).

The commercial H_2_ and CO_2_ gas standard (6 vol. % H_2_, 2 vol. % CO_2_, 92 vol. % N_2_; all gases were 5.0 grade, i.e., 99.999% pure) was obtained pre-mixed and custom-filled in a 1 litre gas cylinder, delivered with an appropriate reducing valve, from Kraiss & Fritz (Stuttgart, Germany). For sampling the commercial H_2_/CO_2_ gas mixture, a 100 mL glass bottle was first evacuated for 2 min (which avoids accumulation of H_2_ at the top of the assembly) using a membrane vacuum pump, then the bottle was flushed with the standard gas mixture for 1 min using the cap assembly (input tubing assembly (2)) shown in Figure 2B. The evacuating and flushing step was repeated once. Then, a dummy 1.5 mL sample was taken via the gas-tight septum and discarded. A second 1.5 mL sample was taken for subsequent measurement in the GC. All measurements were performed in triplicate.

Components required for the construction of the closed setup apparatus used for the H_2_ production experiments were obtained from Bohlender (Grünsfeld, Germany; product brand: BOLA) and Swagelok (Cleveland, OH, USA). The septum used for gas-tight gas sampling was the Septa Thermolite^®^ Shimadzu Plug from Restek (Bad Homburg, Germany). A complete list of the parts used for building the setup apparatus can be found in Supplementary Figure S1 and Supplementary Table S1.

For tight sealing of glass bottles or vials in the gas-dilution experiments, Suba-Seal^®^ rubber septa (Sigma-Aldrich/Merck (Darmstadt, Germany)) were used.

Glass DURAN^®^ bottles and culture flasks were obtained from Schott (Mainz, Germany).

The 2.5 mL Luer-lock gas-tight syringe for gas sampling was obtained from SGE Analytical Science/Trajan Scientific and Medical (Ringwood, Victoria, Australia). The needle used (metal hub; gauge: 23; length: 51 mm; point style: 5 (side port to prevent needle clogging)) was from Hamilton Europe (Giarmata, Romania). Since the syringe was not sample-locked, we determined the kinetics of syringe leakage by removing a sample of fixed volume (1.5 mL) from the sealed H_2_-producing NaBH_4_ reaction bottle (see Figure 2), where the H_2_ level had reached the steady state, and then measuring the sample by gas chromatography after defined syringe waiting times. The loss of gas in the syringe was shown to obey first-order kinetics, allowing us to determine the half-time of gas leakage to be about 140 min. Thus, in the time that is required to take the sample and transfer it to the gas chromatograph loop (less than one minute), we expect a loss of only 0.5% gas sample. This value falls well within the sampling error.

### 4.2. The Gas Chromatograph Protocol

Gases were quantitated by GC using a Thermo Scientific™ TRACE™ 1300 gas chromatograph (Thermo Fisher Scientific, Waltham, MA, USA). The stationary phase consisted of two micropacked columns (packing material: ShinCarbon ST; Silcosteel^®^ coated tubing; outer diameter: 1/16 inch; length: 2 m), connected in series. N_2_ gas (grade 5.0) was used as the mobile phase with an effective flow rate of 10.0 mL/min. Gases were detected using a thermal conductivity detector (TCD), which was maintained at 200 °C during the GC-run (filament temperature: 330 °C; front detector polarity: negative; reference gas: N_2_ (5.0 grade, flow rate: 4.0 mL/min)). Instrument control and data analysis were performed using the Chromeleon 7.2 software package (Thermo Fisher Scientific, Waltham, MA, USA). Gas samples were injected manually, using a gas-tight syringe and a manual injection port at the GC. The GC run was performed at 35 °C for the first 6.5 min. Then, a temperature ramp of 10 °C/min was started from 35 °C to 170 °C until the end of run at 20 min, which was followed by a cooling period to 35 °C that lasted approximately 5 min. Routinely, one or two “dummy” runs with 1.5 mL air were performed prior to gas measurements in order to eliminate spurious signals due to dirt accumulation.

The relevant peak positions of the gas components (see Figure 1) were assigned using ambient air as a standard for O_2_ and CO_2_. The CO_2_ peak position was confirmed by the measurement of a gas sample taken from the headspace of a bottle filled with soda water. With our GC protocol, O_2_ and CO_2_ had a retention time of 3.16 min and 15.76 min, respectively. The retention time of H_2_, as determined using H_2_-containing gas samples, obtained by the reaction of NaBH_4_ with H_2_O, was 1.26 min. An example of a typical GC measurement is shown in Figure 1.

Routinely, the 1 mL sample loop was overfilled with 1.5 mL gas sample, to ensure that no ambient gas enters the loop during measurement. In control experiments, where only 1 mL sample were injected, we observed that about 60 μL of ambient air (6% of the injected volume) was able to enter the measurement loop, thereby raising the standard variation for the O_2_ peak area from 1.4% (overfilled loop) to 2.7%.

The linearity between gas concentration and GC peak area was confirmed by mixing defined volumes of air (which contains 21 vol. % O_2_) with pure N_2_ in the gas-tight 2.5 mL syringe in ratios of 1:0 (21 vol. % O_2_), 2:1 (14 vol. % O_2_), 1:1 (10.5 vol. % O_2_), and 1:2 (7 vol. % O_2_), respectively, and subsequently determining the GC peak areas. All measurements were performed in triplicate. To generate samples with very low O_2_ concentrations (0.5–4 vol. % O_2_), a 17 mL N_2_-filled Pyrex tube (sealed with a Suba-Seal^®^ cap) was “spiked” with varying volumes of air, from which 1.5 mL samples were analyzed by GC. In the event, the peak area was shown to be linear with the [O_2_], although small deviations (probably due to dilution inaccuracies) were observed at the lowest [O_2_].

#### Considerations for the Effect of Elevated Pressure in Gas Samples upon the GC Measurement of Gas Concentrations

When gas samples of any given pressure are injected into the GC sample loop, they immediately equilibrate to atmospheric pressure. Whereas the relative gas composition in the sample remains unaffected, the absolute molar concentration of gas is reduced relative to the initial pressurized sample. This complicates the interpretation of GC measurements from biological experiments where the samples taken are often at elevated pressure (e.g., for the sampling of only a gas head space of 2–3 mL present in a 120 mL-culture bottle filled with a photosynthetically growing anaerobic *R. rubrum* culture, from which gas samples are taken).

Attempts to dilute high pressure gas samples with N_2_ directly in the gas-tight syringe were not successful, since unacceptably large errors occurred. Therefore, for high pressure samples we devised an alternative methodology, in which the gas sample (usually 0.5–1.0 mL) was injected into a 260.9 mL gas dilution bottle (sealed with a Suba-Seal^®^ cap). This approximately 250-fold dilution eliminates the pressure effect, and 1.5 mL samples taken from the gas dilution bottle for GC-measurement can be regarded as samples at atmospheric pressure.

### 4.3. BOLA and Swagelok Components and Connections used for Constructing Gas Sampling Assemblies

To make gas-tight connections between lab glassware bottles and flasks, a screw joint system is required which connects the thread on the glassware (usually of “GL”-type) with a screw cap containing the tubing. In the commercially available (BOLA) screw joint system described here, three different sealing rings mesh perfectly, so that the cap with the tubing is screwed tightly onto the GL thread. The same screw joint sealing methodology is also used in technical systems (here: Swagelok), in order to connect metal parts with threads to tubes via tube fittings. The threads in technical systems are usually of NPT or ISO type. Screw joints provide much more flexibility in comparison to setups, which were made gas-tight by welding the parts together.

Most of the laboratory screw joint system and other components for EX-protection (e.g., tube-fittings, tubing, and stopcocks) used here were standard BOLA parts (GL-14 thread size, tubing with 6 mm outer diameter and 4 mm inner diameter). The coupling joint, however, containing a GL-14 inner thread and a cylindrical G1/4” inner thread, which connected the plastic BOLA-parts with the metal Swagelok septum assembly (see Figure 2B), was a special model (EX-protected) manufactured for us by Bohlender. Gas-tightness of the connections was tested by flushing the setup with N_2_ at high pressure and checking for foam formation by using a commercial tenside formulation (the Snoop^®^ liquid leak detector (Swagelok)) which is commonly used in engineering environments for the detection of very small leaks. Even though the threads were well-fitting, it was difficult to make gas-tight connections between the plastic and the metal parts. Only after fixing this connection with three different gasket rings (from Swagelok), gas-tightness could be achieved. The whole assembly containing the three-gasket connection setup was shown to be vacuum-tight for a period of several days in preliminary growth experiments where only a 1 litre bottle was used as the gas reservoir instead of the two 5 litre bottles used later (Figure 4). In the preliminary experiment, a vacuum developed, which prevented sampling using a gas-tight syringe.

The septum assembly was made of metal Swagelok parts, because the septum used fits perfectly into the 1/8” Swagelok tube fitting at the end of the assembly. This septum assembly was gas-tight, even after autoclaving and several sampling events (all calibration experiments could be performed using the same septum). For the bacterial growth experiments, however, we regularly used a new septum as precaution against septum fatigue during the course of the experiment.

Finally, we note that, whereas the connections of the plastic-type apparatus described above are straightforward, the connections between steel and plastic tubing often require some level of improvisation (e.g., more than one intermediate washer (gasket) at the steel-plastic interface, or attention to the compatibility of “cylindrical” or “conical” screw connections, etc.) in order to achieve a high level of gas-tightness.

### 4.4. Calibration of the H_2_ GC-Signal

Justification for Equations (6)–(10) in Section 2:

In the closed bottle in the absence of NaBH_4_-induced H_2_, we can write the gas pressures with Dalton’s law (see Section 2 for symbol definitions):

(11) $$P_{T}V=(P_{O_{2}}+P_{N_{2}}+P_{CO_{2}}+\sum_{i}P_{i})V$$

which can also be written as:

(12) $$P_{T}\left(air\right)=n_{o_{2}}\frac{RT}{V}+n_{N_{2}}\frac{RT}{V}+n_{CO_{2}}\frac{RT}{V}+\underset{i}{\sum }n_{i}\frac{RT}{V}$$

In the presence of NaBH_4_-induced H_2_, the total pressure increases to $P_{T}^{H}$, and Equation (12) is modified:

(13) $$\;\;\;P_{T}^{H}\left(air+H_{2}\right)=n_{H_{2}}\frac{RT}{V}+n_{o_{2}}\frac{RT}{V}+n_{N_{2}}\frac{RT}{V}+n_{CO_{2}}\frac{RT}{V}+\underset{i}{\sum }n_{i}\frac{RT}{V}$$

If 1 mL of this gas is filled in the GC sample loop, $P_{T}^{H}$ will once again be reduced to P_T_ by a factor γ:

(14) $$P_{T}\left(air+H_{2}\right)=\gamma^{-1}\;P_{T}^{H}=\frac{RT}{V}\left(\gamma^{-1}n_{H_{2}}+\gamma^{-1}n_{O_{2}}+\gamma^{-1}n_{N_{2}}+\gamma^{-1}n_{CO_{2}}+\gamma^{-1}\sum_{i}n_{i}\right)$$

or

(15) $$P_{T}\left(air+H_{2}\right)=\gamma^{-1}\;P_{T}^{H}=\frac{RT}{V}\left(n\prime_{H_{2}}+n\prime_{O_{2}}+n\prime_{N_{2}}+n\prime_{CO_{2}}+\sum_{i}{n_{i}}^{\prime }\right)$$

Note that our formalism uses only the ideal gas equation and does not require corrections for real gases. The rationale for this assumption is that only ambient or near ambient pressures are relevant in most physiological experiments. For mesobaric organisms, excess pressures above about 1.5 atm are severely inhibitory for growth, so that the real gas corrections are essentially negligible. We illustrate this using a simple calculation. Considering only oxygen, the deviation from ideal gas behaviour can be described using the virial equation [30]:

(16) $$PV_{m}=RT\left(1+\frac{B}{V_{m}}+\frac{C}{V_{m}^{2}}+\frac{D}{V_{m}^{3}}+\ldots \right)$$

where V_m_ is the molar volume of the gas component and the constants in parentheses (1, B, C, etc.) are the so-called virial coefficients. At near-ambient pressures, only the second virial coefficient, B, is numerically significant, so that Equation (16) simplifies to:

(17) $$PV_{m}=RT\left(1+\frac{B}{V_{m}}\right)$$

Thus, the deviation from ideal gas behaviour is given by the deviation in parentheses. For oxygen, V_m_ at 298.15 K (25 °C) and 1 atm pressure is 24,465 cm^3^·mol^−1^, and B (at 298.15 K) is −16.4118 cm^3^·mol^−1^ [30]. Thus, the value of B/V_m_ is 6.7 × 10^−4^, which corresponds to a −0.07% deviation from the ideal gas equation. For N_2_ and H_2_, the equivalent deviations from the ideal gas equation are also very small (about −0.022%, and +0.05%, respectively). For CO_2_, where B is significantly larger (−124 cm^3^·mol^−1^) than for the aforementioned gases, the deviation from ideal gas behaviour corresponds to about 0.5%, which is still well within the experimental accuracy of sample measurement (about 5%). Thus, the assumption of ideal gas behaviour is sufficiently accurate for most conditions in a physiological context.

### 4.5. Bacterial Strain, Growth Conditions, and Spectroscopic and Biochemical Analysis of Growth Parameters

The *R. rubrum* wild-type strain S1 [31] was used for the growth experiments. Semi-aerobic (P_O2_ < 0.3% [21]), dark cultivation of the pre-culture was performed in a 250 mL baffled Erlenmeyer flask in a final volume of 100 mL modified Sistrom medium, M2S [32,33]. For the H_2_-production experiment described here, cells were cultivated in a 1 litre Erlenmeyer flask containing 504 mL M2SF medium, which contains higher concentrations of succinate (40 mM) and fructose (16.7 mM) to enhance the expression of photosynthetic genes, as described [15,21]. The experiment was initiated by inoculating the M2SF culture medium with 20 mL of fully grown *R. rubrum* cells (under dim green light), and after closing the gas-tight access to the flask, commencing incubation in a shaker held in the dark at 30 °C. Flasks were shaken at 150 rpm (2.5 cm throw; Model G25 Incubator Shaker, New Brunswick Scientific Co. Inc., Edison, NJ, USA).

For absorption measurements and fructose determinations during the growth curve, 2 mL samples were taken, twice a day, with a sterile plastic syringe via the liquid sampling tube (see Figure 4). Before sampling, approximately 5 mL of the culture, residing inside the tube, were taken and discarded. The absorption at 660 nm and 882 nm of the culture was measured in 1 mL plastic cuvettes (4 mm path-length) with a LKB-Pharmacia photometer. Sampling was performed under dim green light.

For fructose determination, at each time point, 1 mL of culture was pelleted by centrifuging for 2 min at 14,000 rpm (11,000× *g*), at room temperature, and the medium supernatant stored at −20 °C until required for the determination of fructose. To remove proteins from the medium supernatant, 250 µl medium supernatant were precipitated with 17 µL of a 72% (*w*/*v*) trichloroacetic acid solution (TCA: final concentration: 4.6% (*w*/*v*)), incubated for 30 min at room temperature, and the protein pellet removed by a 20 min centrifugation step (11,000× *g*). The fructose content in 10 µL to 20 µL aliquots of the deproteinized supernatant was determined by performing the modified Kulka method as described in detail by Shaw and Ghosh [34].

The concentrations of gases in the total 12.5 litre gas reservoir of the closed hydrogen producing (524 mL) culture were determined by sampling 1.5 mL with a gas-tight syringe using the sampling port, with subsequent measurement by GC. The molar concentrations were calculated using the GC-conversion coefficients described above. The total sampling volume (sum of all 9 measurements) corresponds to only 0.1% of the total reservoir gas volume, which we consider to be negligible.

## 5. Conclusions

The modular construction system described for performing bio-H_2_ production experiments at the small laboratory scale enables precise gas measurements with a high level of safety. Our protocol for calculating gas concentrations directly from GC measurements is sufficient for many studies, and largely obviates the need for expensive standard pressurized gas mixtures for use as standards. Using this apparatus, we demonstrated that gas production (in particular, H_2_ production) and gas consumption of the purple photosynthetic bacterium, *R. rubrum*, during “dark-photosynthetic” semi-aerobic growth, can be analysed. The methodology described can easily be extended to other systems where gas metabolism is an issue.

## Data Availability

No new data were created or analyzed in this study. Data sharing is not applicable to this article.

## Supplementary Materials

The following are available online at https://www.mdpi.com/article/10.3390/metabo11100667/s1, Figure S1: The gas-tight bottle experimental setups used in this study, Table S1: Part-list.
