# Supplementary material for: New Approach for the Construction and Calibration of Gas-Tight Setups for Biohydrogen Production at the Small Laboratory Scale
Source: Metabolites. 2021 Sep 29;11(10):667. doi: 10.3390/metabo11100667 (PMC8541310; doi:10.3390/metabo11100667)
Supplement: Supplementary file 1 [file metabolites-11-00667-s001.zip › metabolites-1348256-supplementary.pdf]

Supplementary Material for the manuscript:

NEW APPROACH FOR THE CONSTRUCTION AND CALIBRATION OF GAS-TIGHT APPARATUS FOR BIOHYDROGEN PRODUCTION AT THE SMALL LABORATORY SCALE

by: Caroline Autenrieth, Shreya Shaw and Robin Ghosh

Supplementary Figure S1 and Table S1:

The gas-tight bottle experimental setups shown in Figure 2B and Figure 4 are shown in Figure S1A and B, respectively, together with the respective part numbers, which are described in Table S1: *[1,2]

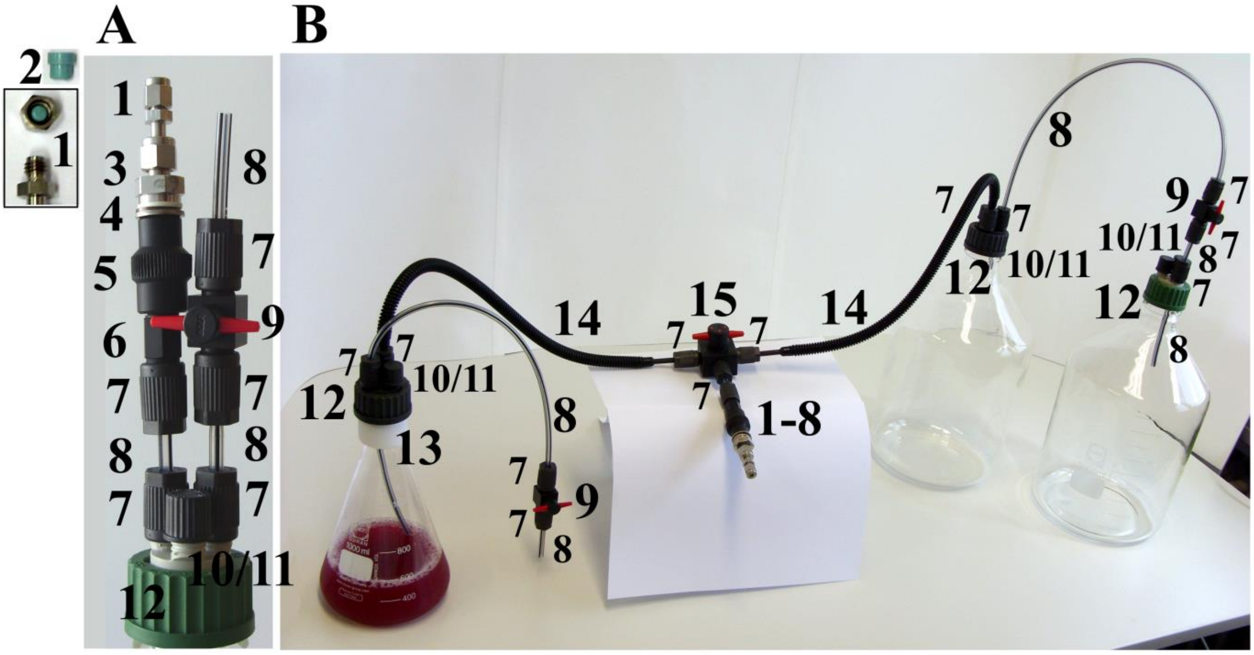

Supplementary Figure S1

Table S1:

No.	Part name	Description	Company	Catalogue Part No. *[3]
1	Septum assembly	Swagelok Tube Fitting, Reducer, 1/8" Tube OD x 1/4" Swagelok Tube Adapter; the 1/8" nut (head piece) holds the Septum; material: stainless steel	Swagelok	SS-200-R-4
2	Septum	Thermolite® Septa (diameter: Shimadzu Plug; fits tightly into stainless steel nut for 1/8" Swagelok tube fitting (Part No. 1))	Restek	27154
3	Connection to GL-system	Swagelok Tube Fitting, Male Connector, 1/4" Tube OD x 1/4" Male ISO Parallel Thread, Straight Shoulder; connects the 1/4" tube from the septum assembly and the plastic part No. 5; material: stainless steel	Swagelok	SS-400-1-4RS
4	Gaskets and O-rings	Different gaskets used to seal the 1/4" connection to the plastic part No. 5; materials: copper, stainless steel/fluorocarbon	Swagelok	CU-4-RP-2<br>SS-4-RS-2V<br>CU-4-RG-2
5	Coupling Joint	Connection containing a cylindrical G1/4" inner thread and a GL-14 inner thread; material: polytetrafluoroethylene (PTFE) with carbon, static dissipative (PTFE-EX)	Bohlender, brand: BOLA	Custom-made
6	GL Tube Fitting EX, straight	Straight tube fitting, two connections with GL-14 thread; material: PTFE-EX	Bohlender, brand: BOLA	D 856-14
7	Laboratory Screw Joint EX	Connects fittings with GL-14 threads with hard-walled tubing, for tubing outer diameter (O.D.): 6 mm; material: screw cap: conductive polyphenylene	Bohlender, brand: BOLA	D 840-74

		sulfide with glass fibres (PPS-EX), inner parts: PTFE-EX		
8	Zebra Explosion-Proof Tubing	Transparent tubing with black longitudinal conductive stripes on the outer surface; O.D.: 6 mm, inner diameter (I.D.): 4 mm, material: tetrafluoroethylene-perfluoro(propyl/vinyl/ether) copolymer (PFA EX)	Bohlender, brand: BOLA	S 1855-40
9	GL Two-Way Stopcock EX	Two-way stopcock with straight bore (4 mm diameter) and two connections with GL-14 thread; material: PTFE-EX	Bohlender, brand: BOLA	E 712-14
10	HT Screw Cap	Screw cap for GL-14 thread, high thermal resistance; material: PPS with integrated PTFE-membrane gasket	Bohlender, brand: BOLA	H 994-14
11	Gasket for caps	Gasket used for tight sealing of GL-14 screw caps (and the connection between No. 5 and 6*[4]); material: lower side: silicone-elastomer, upper side: PTFE.	Bohlender, brand: BOLA	H 973-14
12	Multiple Distributor for Bottles EX	Black conductive screw cap for bottle thread GL 45 (material: PPS-EX), distributor body with 3 GL-14 threaded necks (material: PTFE-EX)	Bohlender, brand: BOLA	D 865-08
13	Threaded Adaptor	Adaptor for the transition from GL 32 (on Duran culture flask) to GL 45 distributor cap; material: PTFE	Bohlender, brand: BOLA	H 978-30
14	Flexible Tubing EX	Conductive corrugated tubing with cylindrical tubing ends (O.D.: 6 mm, I.D.: 4 mm) which can be connected with laboratory screw joints, length: 0.5 m; material: PFA-EX	Bohlender, brand: BOLA	S 1824-24
15	GL Three-Way	Three-way stopcock, T-shaped bore	Bohlender,	E 716-14

	Stopcock EX	(diameter: 4 mm) and three connections with GL-14 thread; material: PTFE-EX	brand: BOLA	

*[1]: For the purpose of the experiments described in this manuscript, the assembly of the Swagelok-parts is probably too complex for many applications. The Swagelok-parts had been assembled for a flexible technical setup (which will be described elsewhere) and we adjusted it to the BOLA-parts as described. An easier way to connect the septum with the BOLA-parts would be using the following Swagelok part ("A1,3") instead of the parts 1 and 3:

A 1,3	Swagelok Tube Fitting, Male Connector, 1/8" Tube OD x 1/4" Male NPT	Alternative to 1,3; connection 1: 1/8" Swagelok Tube Fitting for insertion of the septum; connection 2: 1/4" male NPT for insertion into Coupling Joint (Part No. 5); material: stainless steel	Swagelok	SS-200-1-4

The connection between this Swagelok tube fitting (A1,3) with the Coupling Joint (Part No. 5) can be sealed with a Teflon tape. The Teflon tape can also be an alternative for the gaskets and O-rings (Part No. 4), because in contrast to an ISO parallel thread, the NPT-thread suggested does not need a gasket, since it is self-sealing.

*[2]: If the setup is assembled for the purpose shown in Supplementary Figures S1A (evacuating and flushing of a bottle, taking gas samples via the septum), the Parts Nos. 1-8 are necessary, since the Coupling Joint (Part No. 5) cannot be connected directly to the bottle because of steric hindrance with the GL-14 screw caps. However, for connection of the septum with the apparatus shown in Figure S1B, the Coupling Joint (Part No. 5) could be directly connected with the Three-Way Stopcock (Part no. 15). (See also footnote *[4] for instructions to seal the connection.)

*[3]: see websites of the respective companies:
https://www.swagelok.de/en/product/fittings/tube-fittings-tube-adapters
https://www.restekgmbh.de/produkte/zubehoer/septa-plug-sri-gcs
https://www.bola.de/en/products/screw-joints-and-components-for-ex-protection/?p=2

*[4]: Gas-tightness tests of the setup (where the setup was placed under a N_2 positive pressure) had revealed, that the connection between the Coupling Joint (Part No. 5) and the GL Tube Fitting (Part No. 6) was not gas-tight. Therefore, a Gasket (Part No. 11; with a central hole (diameter approx. 2 mm) cut out using a needle) was inserted into the inner GL14 threat of the Coupling Joint. This sealed the connection.
